# Silver Nanoparticles from Oregano Leaves’ Extracts as Antimicrobial Components for Non-Infected Hydrogel Contact Lenses

**DOI:** 10.3390/ijms22073539

**Published:** 2021-03-29

**Authors:** Anastasia Meretoudi, Christina N. Banti, Panagiotis K. Raptis, Christina Papachristodoulou, Nikolaos Kourkoumelis, Aris A. Ikiades, Panagiotis Zoumpoulakis, Thomas Mavromoustakos, Sotiris K. Hadjikakou

**Affiliations:** 1 Inorganic Chemistry Laboratory, Department of Chemistry, University of Ioannina, 45110 Ioannina, Greece; ameretoudi1996@gmail.com (A.M.); panagiwtisraptis93@yahoo.gr (P.K.R.); 2 Department of Physics, University of Ioannina, 45110 Ioannina, Greece; xpapaxri@uoi.gr (C.P.); ikiadis@uoi.gr (A.A.I.); 3 Medical Physics Laboratory, Medical School, University of Ioannina, 45110 Ioannina, Greece; nkourkou@uoi.gr; 4 Laboratory of Chemistry, Analysis & Design of Food Processes, Department of Food Science and Technology, University of West Attica, 11635 Attica, Greece; pzoump@eie.gr; 5 Organic Chemistry Laboratory, Department of Chemistry, University of Athens Greece, 15571 Athens, Greece; tmavrom@chem.uoa.gr; 6 Institute of Materials Science and Computing, University Research Center of Ioannina (URCI), 45110 Ioannina, Greece

**Keywords:** silver nanoparticles, oregano leaves’ extract, antimicrobial materials, hydrogels, contact lens

## Abstract

The oregano leaves’ extract (ORLE) was used for the formation of silver nanoparticles (AgNPs(ORLE)). ORLE and AgNPs(ORLE) (2 mg/mL) were dispersed in polymer hydrogels to give the pHEMA@ORLE_2 and pHEMA@AgNPs(ORLE)_2 using hydroxyethyl–methacrylate (HEMA). The materials were characterized by X-ray fluorescence (XRF) spectroscopy, X-ray powder diffraction analysis (XRPD), thermogravimetric differential thermal analysis (TG-DTA), derivative thermogravimetry/differential scanning calorimetry (DTG/DSC), ultraviolet (UV-Vis), and attenuated total reflection mode (ATR-FTIR) spectroscopies in solid state and UV–Vis in solution. The crystallite size value, analyzed with XRPD, was determined at 20 nm. The antimicrobial activity of the materials was investigated against Gram-negative bacterial strains *Pseudomonas aeruginosa* (*P. aeruginosa*) and *Escherichia coli* (*E. coli*). The Gram-positive ones of the genus of *Staphylococcus epidermidis* (*S. epidermidis*) and *Staphylococcus aureus* (*S. aureus*) are known to be involved in microbial keratitis by the means of inhibitory zone (IZ), minimum inhibitory concentration (MIC), and minimum bactericidal concentration (MBC). The IZs, which developed upon incubation of *P. aeruginosa*, *E. coli*, *S. epidermidis,* and *S. aureus* with paper discs soaked in 2 mg/mL of AgNPs(ORLE), were 11.7 ± 0.7, 13.5 ± 1.9, 12.7 ± 1.7, and 14.3 ± 1.7 mm. When the same dose of ORLE was administrated, the IZs were 10.2 ± 0.7, 9.2 ± 0.5, 9.0 ± 0.0, and 9.0 ± 0.0 mm. The percent of bacterial viability when they were incubated over the polymeric hydrogel discs of pHEMA@AgNPs(ORLE)_2 was interestingly low (66.5, 88.3, 77.7, and 59.6%, respectively, against of *P. aeruginosa*, *E. coli*, *S. epidermidis,* and *S. aureus*) and those of pHEMA@ORLE_2 were 89.3, 88.1, 92.8, and 84.6%, respectively. Consequently, pHEMA@AgNPs(ORLE)_2 could be an efficient candidate toward the development of non-infectious contact lenses.

## 1. Introduction

Contact lenses are made of hydrogels such as poly–2–hydroxyethylmethacrylate (pHEMA) [1,2]. The pHEMA is a synthetic biocompatible hydrogel material for contact lens’ development, which was approved by the Food and Drug Administration (FDA) in 1971 [3]. The advantages of the use of pHEMA in contact lenses arise from its water and oxygen permeability [1,2]. However, bacteria such as *Pseudomonas aeruginosa, Escherichia coli, Staphylococcus epidermidis,* and *Staphylococcus aureus* are often colonizing contact lens’ materials [4,5]. Thus, contact lens may be the etiology of bacterial infections such as Microbial Keratitis (MK). This initiates the research for novel material’s’ development, which exhibits the benefits of typical contact lenses without the peril for severe bacterial infections. The dispersion of antimicrobial agents leads to loading of antibacterial properties in the polymeric material [6,7]. Silver(I) ions have been pursued as additives to contact lenses for their antimicrobial efficacy [8]. However, the major drawback of the ionic silver usage is the inactivation through complexation with chloride anions of the human fluids and their precipitation. Silver nanoparticles, on the other hand, are a valuable alternative [8]. The silver nanoparticles show efficient antimicrobial property compared to other salts due to their extremely large surface area, which provides better contact between the nanoparticles and the cells of the microorganisms [9].

While essential oils, such as *Origanum vulgare*, exhibit antibacterial, antifungal, antiviral, anti-inflammatory, and antioxidant properties [10,11], plant extracts are used for chemical synthetic procedure of nanomaterials as reducing or stabilizing agents [12]. The type of the plant extracts affects the characteristics of the produced nanoparticles since different plant extracts contain different concentrations and combinations of organic reducing agents [12]. Thus, AgNPs from ethanolic oregano leaves’ extracts exhibit stronger antimicrobial activity against Gram-positive (*Staphylococcus aureus*, *Bacillus subtilis*) strains than against Gram-negative (*Escherichia coli*, *Salmonella typhimurium*) strains [13]. Moreover, silver nanoparticles from aqueous oregano plant extract have shown antibacterial and antifungal activities against *Shigella sonnei*, *Micrococcus luteus*, *Escherichia coli*, *Aspergillus flavus*, *Alternaria alternate*, *Paecilomyces variotii*, and *Phialophora alba* [14]. AgNPs from essential oil of oregano possessed antimicrobial activity against *S. aureus* [10].

Aiming for the formation of new, non-infectious materials for sterile antimicrobial contact lenses’ development, we report here the synthesis of AgNPs(ORLE) obtained from oregano leaves extract (ORLE). Consequently, they were incorporated in pHEMA hydrogels. The materials pHEMA@ORLE_2 and pHEMA@AgNPs(ORLE)_2 were characterized by XRF, XRPD, TG-DTA, DTG/DSC, UV-Vis, ATR–FTIR, and refractive index in solid state and UV–Vis spectroscopy in solution. The antimicrobial activity of the materials was evaluated against the Gram-negative bacterial strains *P. aeruginosa* and *E. coli* and the Gram-positive ones, *S. epidermidis* and *S. aureus*, which are involved in microbial contamination of contact lenses, leading to ocular infections.

## 2. Results

### 2.1. General Aspects

**Scheme 1:**
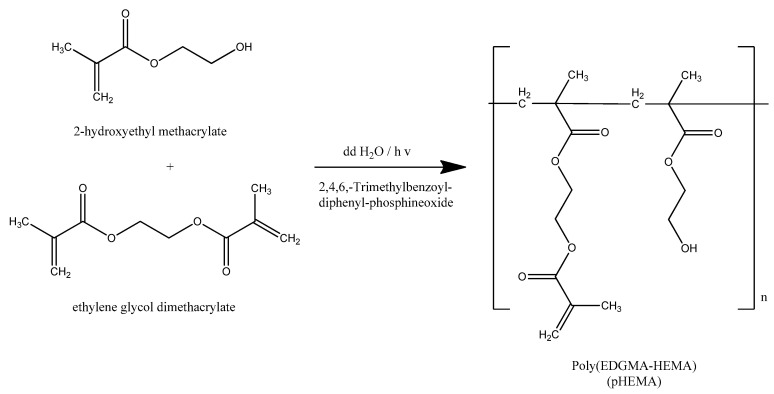
Preparation reaction of poly-hydroxyethyl–methacrylate. (pHEMA).

In order to evaluate the antimicrobial efficiency of contact lens with antimicrobial components, a solution of AgNPs(ORLE) (2 mg/mL) was dispersed in pHEMA (Scheme 1) during its polymerization procedure. Consequently, the pHEMA@AgNPs(ORLE)_2 was derived. Similarly, oregano leaves’ extract (ORLE) was isolated and used for the preparation of pHEMA@ORLE_2. The pHEMA, pHEMA@ORLE_2, and pHEMA@AgNPs(ORLE)_2 discs of 10-mm diameter were cut, cleaned from monomers, and stored either in sterilized NaCl 0.9% *w/w* solution or they were dried at 50 °C (Figure S1).

### 2.2. Solid State

#### 2.2.1. Refractive Index

The refractive indexes of stored discs in saline solution exhibit almost the same refractive index (1.434 (pHEMA), 1.434 (pHEMA@ORLE_2), and 1.433 (pHEMA@AgNPs(ORLE)_2)). These values are in accordance with the corresponding one of the pHEMA@platinum–nanoparticles (1.424–1.436) [8] and pHEMA@AGMNA–1 (AGMNA= {[Ag_6_(*μ_3_*-HMNA)_4_(*μ_3_*-MNA)_2_]^2−^∙[(Et_3_NH)^+^]_2_∙(DMSO)_2_∙(H_2_O)}) (1.436) [7], although the ideal hydrogels should have a refractive index value matching the range of 1.372–1.381 [3]. However, the fabricated hydrogel is highly transparent, with refractive indexes ranging from 1.42 to 1.45 in the spectra range from 400 to 800 nm [15,16].

#### 2.2.2. X-ray Fluorescence Spectroscopy

**Figure 1:**
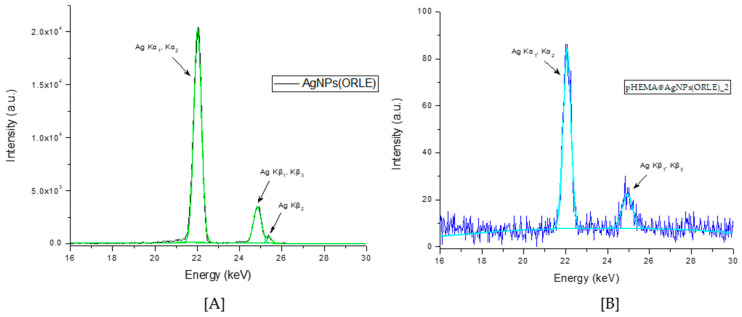
The Ag Kα radiation emitted from AgNPs(ORLE) (**A**) and pHEMA@AgNPs(ORLE)_2 (**B**) Oregano leaves extract (ORLE).

The XRF spectrum of the AgNPs(ORLE) and pHEMA@AgNPs(ORLE)_2 powders confirmed the presence of Ag. Moreover, the Ag Kα X-ray emission was used for quantitative determination of Ag in the AgNPs(ORLE). The content of silver in AgNPs(ORLE) was determined at 37 ± 5% *w/w*. (Figure 1). However, the silver content in the case of pHEMA@AgNPs(ORLE)_2 was unable to be determined due to its low quantity, which was close to the limits of the method.

#### 2.2.3. X-ray Powder Diffraction Analysis (XRPD)

**Figure 2:**
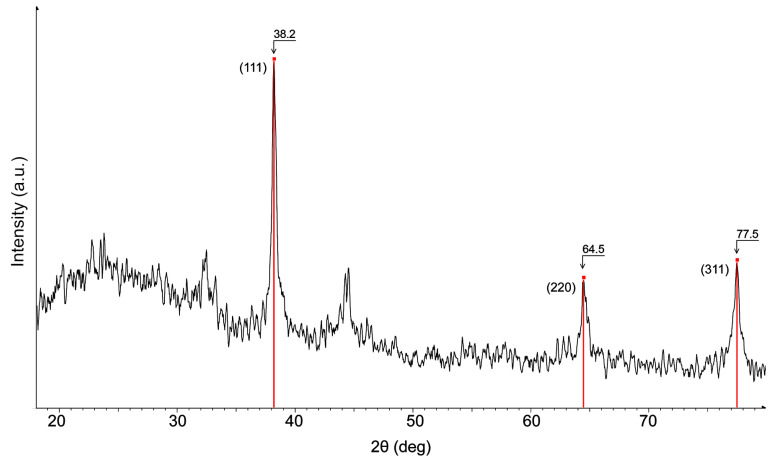
Powder X-ray diagram of sample AgNPs(ORLE) with the characteristic Bragg peaks of Ag nanoparticles.

To perform crystallite size analysis of AgNPs(ORLE) with XRPD (Figure 2), the different contributions to specimen broadening were separated. The observed diffraction profile was the result of the of a specimen profile (f) and the aberrations introduced by the diffractometer. Therefore, the shape of the instrument line profile (g) due to the non-ideal optical contribution of the diffractometer and the wavelength distribution of the radiation should be known. Using a corundum (Al_2_O_3_) standard, the g was convoluted with f, in order to obtain a calculated instrument line profile, which was then applied to the measured profile of our sample (h). The pseudo-Voigt shape function was used, and the curve shape parameter, the skewness, and the kα_2_ contribution were allowed to be refined during the peak fitting. The peak width was defined as the full width at half maximum (FWHM), knowing that the peak width varied inversely with crystallite size. Microstrain broadening analysis was not performed and, therefore, results might be less accurate at larger 2-theta angles. The profiles of the three Bragg peaks at 38.2, 64.5, and 77.5 were calculated and the following crystallite sizes were obtained: 210, 197, and 194 Å. The similar obtained values showed minimal crystalline strain, yielding an average crystallite size value of approximately 20 nm.

#### 2.2.4. Thermogravimetric Analysis of AgNPs(ORLE), pHEMA, and pHEMA@AgNPs(ORLE)_2

##### Differential Scanning Calorimetry (DSC)

**Figure 3:**
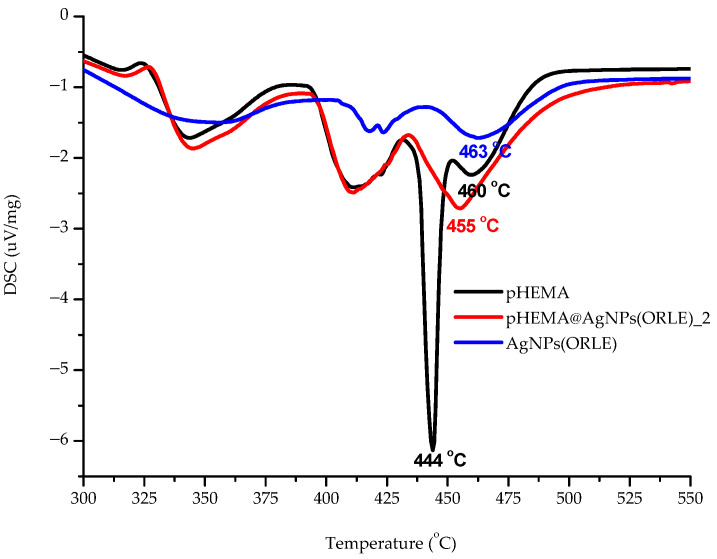
Differential scanning calorimetry (DSC) thermo-diagrams of pHEMA, AgNPs(ORLE), and pHEMA@AgNPs(ORLE)_2.

In order to clarify whether AgNPs(ORLE) interact with pHEMA in solid state leading to a mixture or a composite material, DSC studies were carried out on the powder of dry pHEMA and pHEMA@AgNPs(ORLE)_2 (Figure 3). An endothermic transition at 455.3 °C in the DSC thermo-diagram of pHEMA@AgNPs(ORLE)_2 was observed at higher temperature at 459.7°C in the free pHEMA, suggesting the formation of a composite material rather than a mixture of precursors. The formation of the composite material was further supported by the sharp endothermic transition at 443.8 °C in the diagram of pHEMA@AgNPs(ORLE)_2, which is absent in the corresponding diagrams of AgNPs(ORLE) and pHEMA, respectively (Figure 3).

##### Thermal Decomposition of AgNPs(ORLE) and The Composite pHEMA@AgNPs(ORLE)_2

**Figure 4:**
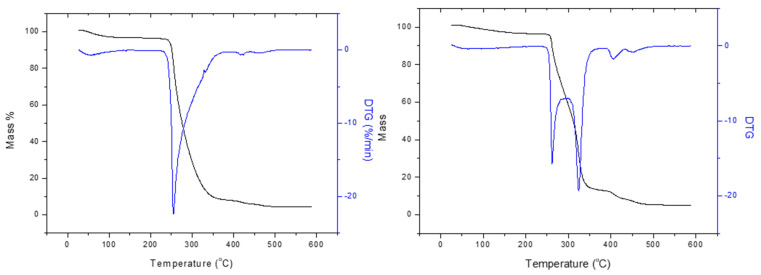
TGA diagrams of AgNPs(ORLE) (**left**) and pHEMA@AgNPs(ORLE)_2 (**right**).

TG-DTA analysis was performed under air on the powder of dry AgNPs(ORLE) and pHEMA@AgNPs(ORLE)_2 with increasing the temperature at a rate of 10 °C min^–1^ from ambient up to 500 °C (Figure 4). The composite pHEMA@AgNPs(ORLE)_2 decomposed with five endothermic steps at 50–221, 221–298, 298–376, 376–430 and 430–527 °C with total mass loss 96.0%. The AgNPs(ORLE) decomposed with four endothermic steps at 50–218, 218–393, 393–424, and 424–463 °C with total mass loss of 96.5% (Figure 4).

#### 2.2.5. Ultraviolet-Visible Spectroscopy (UV–Vis)

##### Solid State UV–Vis Spectra

**Figure 5:**
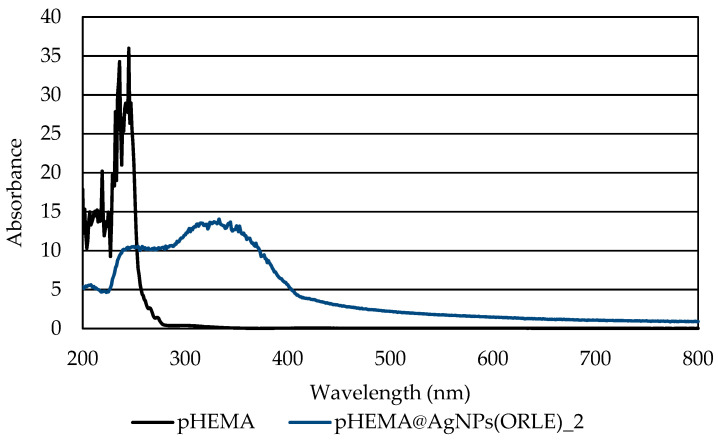
UV solid state of pHEMA and pHEMA@AgNPs(ORLE)_2.

The dispersion of the AgNPs(ORLE) in pHEMA within pHEMA@AgNPs(ORLE)_2 was verified by the solid state UV–Vis spectroscopy (Figure 5). The characteristic broad band at 330 nm in the solid state UV–Vis spectrum of pHEMA@AgNPs(ORLE)_2, which is absent in the corresponding one of free pHEMA, suggested the incorporation of the AgNPs(ORLE) in it.

##### Solution State Spectra

**Figure 6:**
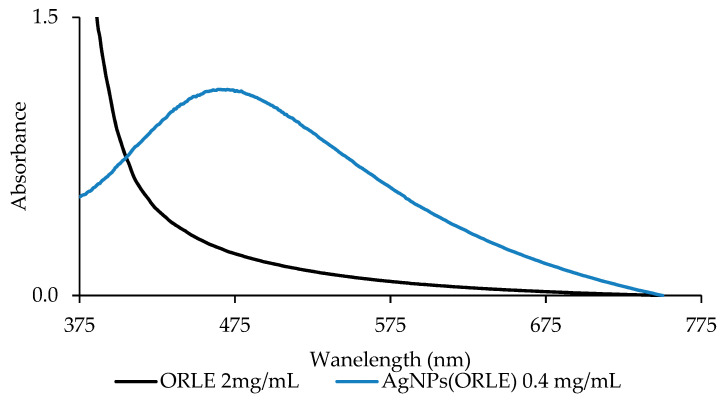
UV–Vis spectrum of AgNPs(ORLE) in double-distilled (dd) water solution.

Since absorption band in UV–Vis spectra of AgNPs between 400–500 nm has been assigned to the collective resonance of electrons at the surface of the silver nanoparticles, these bands were used for the determination of silver nanoparticles’ size [17,18]. The UV–Vis spectrum of AgNPs(ORLE) in double distilled (dd) water solution (0.4 mg/mL) (Figure 6) exhibited a broad band at 479 nm, which correlated to nanoparticles’ size of ~65 nm [19]. It is mentioned here that the size of 20 nm, which was determined by XRPD spectroscopy (see above), refers to crystallite sizes and not to solubilized particles studied here by UV–Vis spectroscopy.

### 2.3. Antibacterial Activity of ORLE and AgNPs(ORLE)

#### 2.3.1. Determination of the Inhibition Zone (IZ) through Agar Disc-Diffusion Method

The agar disc-diffusion method was employed in order to survey the sensitivity of the microorganism to the antibacterial agent studied here [20]. The diameters of the bacterial growth inhibition zones when *P. aeruginosa, E. coli, S. epidermidis,* and *S. aureus* were treated by ORLE and AgNPs(ORLE) with a dose of 2 mg/mL upon their incubation for 20 h are summarized in Table 1 (Figure S2). The bacteria showed higher sensitivity to the AgNPs(ORLE) than ORLE, which rose up to 1.6-fold higher in the case of in the case of *S. aureus.* The microbe strains were classified into three categories according to the size of IZ, caused by an antimicrobial agent in their agar dilution culture: (1) strains, where the agent causes IZ ≥17 mm, were susceptible; (ii) those where the agent created IZ between 13 to 16 mm (13 ≤ IZ ≤ 16 mm) were intermediate; and (3) in those where the agent caused IZ ≤ 12 mm, the microbes were considered as resistant strains [21]. Therefore, the strains studied here (*P. aeruginosa, E. coli, S. epidermidis,* and *S. aureus*) responded intermediately to AgNPs(ORLE), while they were considered as resistant strains toward ORLE [21].

**Figure 7:**
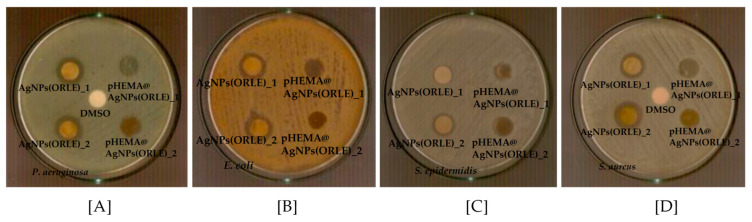
Inhibition zones of pHEMA and pHEMA@AgNPs(ORLE)_2 against *Pseudomonas aeruginosa* (*P. aeruginosa*) (**A**), *Escherichia coli* (*E. coli*) (**B**), *Staphylococcus epidermidis* (*S. epidermidis*) (**C**), and *Staphylococcus aureus* (*S. aureus*) (**D**).

The antimicrobial activity of AgNPs(ORLE) against the microbes prompted us to load it into pHEMA for the development of new non-infectious contact lens pHEMA@AgNPs(ORLE)_2. The pHEMA@ORLE_2 was also prepared for comparison. The inhibition zones of pHEMA@AgNPs(ORLE)_2 against *P. aeruginosa, E. coli, S. epidermidis.* and *S. aureus* microbes suggested mild antimicrobial activity (Table 1, Figure 7). Moreover, no inhibition zones were developed when pHEMA@ORLE_2 or pHEMA was used against all tested strains (Figure S3). Since the agar diffusion test showed that pHEMA@AgNPs(ORLE)_2 discs, with diameter of 10 mm, which contained 2 mg/mL AgNPs(ORLE), developed shorter IZs against *P. aeruginosa, E. coli, S. epidermidis,* and *S. aureus* (10.3 ± 0.7, not developed (ND), 11.0 ± 1.9, and 10.3 ± 0.7 mm, respectively) than soaked paper discs, (diameter of 9 mm) with a solution of AgNPs(ORLE) (2 mg/mL) (13.1 ± 1.6, 12.3 ± 0.7, 12.7 ± 1.7, and 14.8 ± 1.1 mm, respectively), a minor releasing of the discs’ ingredient could be concluded.

**Figure 8:**
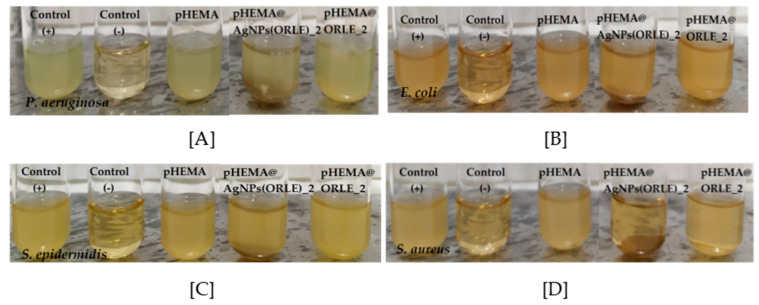
Bacteria viability of pHEMA, pHEMA@ORLE_2, and pHEMA@AgNPs(ORLE)_2 and against *P. aeruginosa* (**A**), *E. coli* (**B**), *S. epidermidis* (**C**), and *S. aureus* (**D**).

Thus, pHEMA, pHEMA@ORLE_2, and pHEMA@AgNPs(ORLE)_2 discs were placed in tests tubes that contained 5 × 10^5^ cfu/mL of *P. aeruginosa, E. coli, S. epidermidis,* and *S. aureus* microbes. The calculated bacterial% viability of *P. aeruginosa, E. coli, S. epidermidis,* and *S. aureus* upon their incubation with pHEMA@AgNPs(ORLE)_2 discs for 20 h was 66.5, 88.3, 77.7, and 59.6%, respectively (Figure 8). On the contrary, no or meaningless influence in the bacterial viability was observed against these bacterial strains, upon their treatment by discs of pHEMA@ORLE_2 or pure pHEMA (Table 1, Figure 8).

#### 2.3.2. Effects on Biofilm Formation

The adhesion of bacterial cells to surfaces is the initial stage of the formation of biofilm. Thus, bacterial colonies are attached in a surface and they are protected in a polysaccharides’ matrix. The necessity of the surgical removal of the infected tissue, which is then followed in the untreated cases, contains high risk for corneal blindness. The biofilm elimination can be achieved by applying metallodrugs [22].

The removal of biofilm was also assessed using crystal violet assay [23]. The discs of pHEMA@AgNPs(ORLE)_2 eliminated the biofilm of *P. aeruginosa* by 22%, while no inhibitory activity was observed against the biofilm of *S. aureus* (Figure S4).

#### 2.3.3. Minimum Inhibitory (MIC) and Minimum Bactericidal (MBC) Concentrations

The antimicrobial potency of ORLE and AgNPs(ORLE) against Gram-negative (*P. aeruginosa* and *E. coli)* and Gram-positive *(S. epidermidis* and *S. aureus*) was evaluated by the mean of MIC, which is the lowest concentration needed for the inhibition of the bacterial growth, and the MBC, which is the lowest concentration of an antibacterial agent that can eliminate 99.9% of the bacterial inoculum [23,24]. The MIC and MBC values of ORLE and AgNPs(ORLE) against microbes studied here are summarized in Table 1 (Figures S5–S10). ORLE was inactive against the strains used.

#### 2.3.4. Effects on Biofilm Formation by AgNPs(ORLE)

The effect of AgNPs(ORLE) against biofilm formation of *P. aeuroginosa* and *S. aureus* was studied by the biofilm elimination concentration (ΒΕC) using crystal violet assay [7,23]. The ΒΕC was defined as the concentration required to achieve at least a 99.9% reduction in the viability of biofilm bacteria. The composite material inhibited the biofilm formation, reducing 100% of its biomass, at 945 ± 72 μg/mL, against *P. aeuroginosa,* while it was not determined for *S aureus* for concentrations up to 1000 μg/mL (Figure S11). The BEC value of ciprofloxacin, a known antibiotic use for the bacterial keratitis, was 221 μg/mL toward *P. aeuroginosa* [6].

#### 2.3.5. In Vitro Toxicity against Normal Human Corneal Epithelial Cells (HCECs)

The in vitro toxicity of ORLE and AgNPs(ORLE) was evaluated against normal human corneal epithelial cells (HCECs) after their incubation for a period of 48 h. The Inhibitory Concentration of the 50% of the cells (IC_50_) values for ORLE and AgNPs(ORLE) were higher than 200 μg/mL, suggesting low toxicity toward normal cells. The cell viability of HCECs upon their incubation with discs of pHEMA@ORLE_2 and pHEMA@AgNPs(ORLE)_2 was decreased at 90.0 ± 9.5 and 54.9 ± 7.1%, respectively, in respect of the cells incubated with pHEMA discs.

#### 2.3.6. In Vivo Toxicity Evaluation by Brine Shrimp Artemia Salina

*Artemia salina* is a zooplanktonic crustacean [25,26] and the nauplii of the brine shrimp are considered as a simple and suitable model system for acute toxicity tests [26,27]. The assay can be correlated with the toxicity data of rodents and humans and shows a good correlation with cytotoxicity tests, making these measurements suitable as preliminary results [26,28,29]. The lethality was noted in terms of deaths of larvae.

The (%) mortality of *Artemia salina* larvae upon their incubation with ORLE and AgNPs(ORLE) at the concentrations of 150–300 μg/mL are summarized in Table 2. The mortality rate (%) of brine shrimp larvae incubated with ORLE and AgNPs(ORLE) rose from 4.0 and 7.1% (150 μg/mL) up to 78% at 300 μg/mL for both ORLE and AgNPs(ORLE) (Table 2). No mortality of brine shrimp larvae was found upon incubation of pHEMA, pHEMA@ORLE_2, and pHEMA@AgNPs(ORLE)_2 for 24 h, indicating their non-toxic behavior.

## 3. Materials and Methods

### 3.1. Materials and Instruments

All solvents used were of reagent grade. Tryptone and soy peptone were purchased from Biolife, Milano, Italy. Agar was purchased from Sigma-Aldrich St. Louis, MO, USA product of Spain. Sodium cloride, D(+)–Glucose, and di potassium hydrogen phosphate trihydrate were purchased from Merck, Darmstadt, Germany. DMSO was purchased from Riedel–de Haen (Seelze, Germany). Melting points were measured in open tubes with a Stuart Scientific apparatus and are uncorrected. A UV–1600 PC series spectrophotometer of VWR international GmbH, Darmstadt, Germany was used to obtain electronic absorption spectra. ATR-FTIR spectra in the region of 4000–370 cm^–1^ were obtained with a Cary 670 FTIR spectrometer, Agilent Technologies Agilent Technologies. XRF measurement was carried out using an Am–241 radio isotopic source (exciting radiation 59.5 keV).

### 3.2. Preparation of ORLE and AgNPs(ORLE)

Dry oregano leaves (4 g) were refluxed using a Soxhlet apparatus with 50 mL dd water for 3 h. The ORLE extract was then obtained with filtration of the initial extract using Whatman No. 1 filter paper. In a beaker (250 mL), 10 mL ORLE and 170 mg AgNO_3_ (1 mmol) were added. The beaker was placed in a microwave oven (700 W) for 2 min. Consequently, the solution was placed in the sonicator (ultrasonic) for 10 min. The solution was centrifugal at 6000 rpm for 20 min and the AgNPs(ORLE) were collected.

ORLE: yield 0.2 g, brown powder, melting point: 180–184 °C (change of color); ATR-IR (cm^–1^): 3855 w, 3265 w, 1590 m, 1427, 1266 w, 1079, 1026, 989, 730, 685, 605, 564, 526, 505, 476, 455, and 426; UV–Vis (ddH_2_O): λmax = 356 nm.

AgNPs(ORLE): yield 0.0232 g; content of Ag in AgNPs(ORLE): 37 ± 5% *w/w*; melting point: >250 °C IR (cm^–1^): 3135 w, 2641, 2324 w, 1699 w, 1594 m, 1504 w, 1442 w, 1225, 1145, 1011, 807 w, 766 w, 7368 w, 692 w, 664 w, 630 w, 595 w, 570 w, 543 w, 509 w, 487, 473 w, 448, 431 s, and 410 s; UV–Vis (ddH_2_O): λmax = 479 nm.

### 3.3. Synthesis of pHEMA@ORLE_2 and pHEMA@AgNPs(ORLE)_2

Hydrogels of ORLE or AgNPs(ORLE) dispersed in pHEMA were obtained as follows: 2.7 mL of HEMA were mixed with 2 mL of double-distilled water (ddw), which contained ORLE or AgNPs(ORLE) (2 mg/mL) and 10 µL of ethyleneglycol dimethacrylate (EGDMA). The solution was then degassed by bubbling with nitrogen for 15 min. Trimethylbenzoyldiphenyl-phosphineoxide (TPO) initiator (6 mg) was added to the solution and mixed for 5 min at 800 rpm. The solution was poured into the mold and was then placed under a UV mercury lamp (λmax = 280 nm), 15 watts, for photopolymerization for 40 min. Unreacted monomers were removed by immersing the gel in water for 15 min. Discs with 10-mm diameter were cut and were washed by immersion in water, NaCl 0.9%, and HCl 0.1 M, and again in water. The discs were then dried at 40 °C until no weight change would occur. The yield of dry pHEMA, pHEMA@ORLE_2, and pHEMA@AgNPs(ORLE)_2 was 1.669 and 1.683 g, respectively.

The pHEMA@ORLE_2: light-brown color; IR (cm^–1^): 624 w, 480 w, 428 w, UV–Vis (solid state): λmax = 237 nm

The pHEMA@AgNPs(ORLE)_2: dark-brown color; Ag content in pHEMA@AgNPs(ORLE): 0.21 ± 0.04% *w/w*; IR (cm^–1^) 624 w, 511 m, 480 w, 424 w; UV–Vis (solid phase): λmax = 332 nm.

### 3.4. Refractive Indexes

The values of refractive indexes of the lenses were measured with an Abbe refractometer (NAR–1T, Atago Co., Ltd., Tokyo, Japan) at 24 °C.

### 3.5. X-ray Fluorescence Spectroscopy

XRF measurement was carried out using an Am–241 radioisotopic source (exciting radiation 59.5 keV). For the detection of X-ray fluorescence, a Si (Li) detector was used. The measuring time was chosen so as to collect ~2000 data on the weaker Kα peak.

### 3.6. X-ray Powder Diffraction (XRPD)

The study of the samples by using the X-ray powder diffraction was accomplished by a diffraction meter D8 AdvanceBruker, department of Physics, University of Ioannina. Radiation CuKa (40 kV, 40 mA, λKα) and the monochromator system of diffracted beam were used. The X-ray powder diffraction patterns were measured in the area of 2θ angles between 2° and 80°, using a rotation step 0.02° and time of 2 sec per step. All samples measured with the above diffraction meter were in fine-grained powder form.

### 3.7. Thermogravimetric Differential Thermal Analysis (TG-DTA), Differential Scanning Calorimetry (DTG/DSC)

For the DTA/TG measurements, a DTG/TG NETZSCH STA 449C was used. For the measurements, the samples were placed inside a platinum capsule on one side of the thermal scale while on the other side a-alumina was used as reference sample. The speed of temperature increase was 10 °C/min at a temperature range of 25–500 °C and the measurements took place in the air. All the measured samples were in fine-grained powder form.

### 3.8. Bacterial Strains

The bacterial strains of *P. aeruginosa* (*PAO1*), *Escherichia coli* Dh5a (*E. coli*), *S. aureus* (ATCC^®^ 25923™), and *S. epidermidis* (ATCC^®^ 14990™) were adopted in the experiments. The bacterial strain *P. aeruginosa PAO1* was kindly offered from Prof. A. Koukou (Laboratory of Biochemistry, University of Ioannina-Greece). The biological experiments were performed in triplicates. The values were evaluated by the statistical analysis software package included in the MS Office excel.

### 3.9. Effects on the Growth of Microbial Strains

The procedure was performed as previously reported [6,7,23]. Briefly, bacterial strains plated onto trypticase soy agar were incubated at 37 °C for 18–24 h. Three to five isolated colonies of the same morphological appearance were selected from a fresh agar plate using a sterile loop and transferred into a tube containing 2 mL of sterile saline solution. The optical density at 620 nm was adjusted to 0.1, which corresponded to 10^8^ cfu/mL [6,7,23].

For the evaluation of MIC, the inoculum size for broth dilution was 5 × 10^5^ cfu/mL. The culture solution was treated with ORLE and AgNPs(ORLE) (20–300 μg/mL).

For the evaluation of MBC, the bacteria were initially cultivated in the presence of ORLE and AgNPs(ORLE) in broth culture for 20 h. The MBC values were determined in duplicate, by subculturing 4 μL of the broth an agar plate [6,7,23].

In order to evaluate the viability of microbes on pHEMA, pHEMA@ORLE_2, and pHEMA@AgNPs(ORLE)_2, discs of the materials were placed in the test tubes, which contained 5 × 10^5^ cfu/mL of *P. aeruginosa*, *E. coli*, *S. epidermidis,* and *S. aureus* microbes [6,7,23]. The optical densities of the supernatant solutions were then measured to give the % viability of microbes after incubation for 18–24 h [6,7,23].

### 3.10. Removal of Biofilm, Using Crystal Violet Assay

Bacteria with a density of 1.3 × 10^6^ cfu/mL were inoculated into Luria–Bertani agar (LB agar) medium for *P. aeruginosa* or tryptic soy broth for *S. aureus* (total volume = 1500 μL) and cultured for 20 h at 37 °C. Afterwards, the content of each tube was carefully removed, the tubes were washed with 1 mL 0.9% saline dilution, and 2 mL of broth were added. The negative control contained only broth. Then, the bacteria were incubated with ORLE, AgNPs(ORLE), pHEMA, pHEMA@ORLE_2, and pHEMA@AgNPs(ORLE)_2 for 20 h at 37 °C. The content of each tube was aspirated and was washed three times with 1 mL methanol and 2 mL 0.9% saline and left to dry. Then, the tubes were stained for 15 min with crystal violet solution (0.1% *w*/*v*). Excess stain was rinsed off with 1 mL methanol and 2 mL 0.9% saline solution and, afterwards, 3 mL 0.9% saline solution. The tubes were left to dry for 24 h and the bounded crystal violet was released by adding 30% glacial acetic acid. The optical density of the solution yielded was then measured at 550 nm, to give the biofilm biomass [6,7,23].

### 3.11. Determination of the Inhibition Zone (IZ)

The procedure was performed as previously reported [6,7,23]. Agar plates were inoculated with a standardized inoculum (10^8^ cfu/mL) of the microorganisms. Discs of pHEMA or pHEMA@ORLE_2 and pHEMA@AgNPs(ORLE)_2 with 10-mm diameter or ORLE, AgNPs(ORLE) were placed on the agar surface and the Petri plates were incubated for 20 h.

### 3.12. Sulforhodamine B Assay

Initially, the HCECs were seeded in a 24-well plate in a density of 7.5 × 10^4^ cells. After 24 h of cell incubation, ORLE, AgNPs(ORLE), the discs of pHEMA, pHEMA@ORLE_2, and pHEMA@AgNPs(ORLE)_2 were added in the wells. After 24-h incubation of HCECs with the discs, the discs were removed, the culture medium was aspirated, and the cells were fixed with 300 μL of 10% cold trichloroacetic acid (TCA). The plate was left for 30 min at 4 °C, washed five times with deionized water, and left to dry at room temperature for at least 24 h. Subsequently, 300 μL of 0.4% (*w*/*v*) sulforhodamine B (SRB) (Sigma) in 1% acetic acid solution was added to each well and left at room temperature for 20 min. SRB was removed, and the plate was washed five times with 1% acetic acid before air drying. Bound SRB was solubilized with 1 mL of 10 mM unbuffered Tris-base solution. Absorbance was read in a 24-well plate reader at 540 nm [6,7,23].

### 3.13. Evaluation of Toxicity with Brine Shrimp Assay

Brine shrimp assay was performed by a method previously described [3,7,26]. One-g cysts were initially hydrated in freshwater for one hour in a separating funnel or cone-shaped container. Seawater was prepared by dissolving 17 g of sea salt in 500 mL of distilled water [7,26,30]. The container was facilitated with good aeration for 48 h at room temperature and under continuous illumination. After hatching, nauplii released from the eggshells were collected at the bright side of the cone (near the light source) by using a micropipette. The larvae were isolated from the eggs by aliquoting them in a small beaker containing NaCl 0.9% [7,26,30]. An aliquot (0.1 mL) containing about 6 to 10 nauplii was introduced to each well of a 24-well plate and ORLE, AgNPs(ORLE), or one disc of pHEMA or pHEMA@ORLE_2 and pHEMA@AgNPs(ORLE)_2 were added in each well. The final volume of each well was 1 mL with NaCl 0.9%. The brine shrimps were observed at the interval time of 24 h, using a stereoscope. Larvae were considered dead if they did not exhibit any internal or external movement in 10 s of observation. Each experiment was repeated three times.

## 4. Conclusions

This work aimed for the development of sterilized and non-infectious contact lens. For this purpose, oregano leaves’ extract (ORLE) was used for the formation of silver nanoparticles AgNPs(ORLE). AgNPs(ORLE) is an efficient disinfectant. The microbial strains *P. aeruginosa, E. coli, S. epidermidis,* and *S. aureus* involved in microbial keratitis responded intermediately to AgNPs(ORLE). The dispersion of AgNPs(ORLE) in pHEMA resulted in the composite material pHEMA@AgNPs(ORLE)_2. The percent of bacterial viability of *P. aeruginosa, E. coli, S. epidermidis,* and *S. aureus* upon their incubation with pHEMA@AgNPs(ORLE)_2 discs decreased by 33.5, 11.7, 22.3, and 40.4%, respectively, while it was also preventing the formation of biofilm on their surface. The cell viability of HCECA upon their incubation with pHEMA@AgNPs(ORLE)_2 discs for 48 h decreased by 54.9 ± 7.1%. No mortality of brine shrimp larvae was found upon their incubation with pHEMA@AgNPs(ORLE)_2 for 24 h, indicating non-toxic in vivo behavior. Thus, pHEMA@AgNPs(ORLE)_2 is an effective candidate for the development of new non-infectious contact lens.

## Figures and Tables

**Table 1:** Inhibition Zones (IZs). Minimum Inhibitory Concentrations (MICs), Minimum Bactericidal Concentrations (MBCs), Biofilm Elimination Concentrations (ΒΕCs) of ORLE, and AgNPs(ORLE) against *P. aeruginosa, E. coli, S. epidermidis,* and *S. aureus*.

Material Title	*P. aeuroginosa*	*E. coli*	*S. epidermidis*	*S. aureus*
	**IZ (mm)**			
AgNPs(ORLE) 2 mg/mL	13.1 ± 1.6	12.3 ± 0.7	12.7 ± 1.7	14.8 ± 1.1
ORLE 2 mg/mL	10.2 ± 0.7	9.3 ± 0.5	ND	ND
pHEMA@AgNPs(ORLE)_2	10.3 ± 0.7	ND	11.0 ± 1.9	10.3 ± 0.7
pHEMA@ORLE_2	ND	ND	ND	ND
	**Bacteria Viability (%)**			
pHEMA@AgNPs(ORLE)_2	66.5	88.3	77.7	59.6
pHEMA@ORLE_2	89.3	88.1	92.8	84.6
	**MIC (μg/mL)**			
AgNPs(ORLE)	139.5 ± 17.4	124.3 ± 12.9	272.2 ± 14.1	>300
ORLE	>300	>300	>300	>300
	**MBC (μg/mL)**			
AgNPs(ORLE)	135.7 ± 35.2	>300	>300	>300
ORLE	>300	>300	>300	>300
	**BEC (μg/mL)**			
AgNPs(ORLE)	945 ± 72	–	–	>1000

ND = Not Developed (IZ) was developed. ORLE = oregano leaves extract. IZ= Inhibition Zone. MIC= Minimum Inhibitory Concentration, MBC= Minimum Bactericidal Concentration, BEC= Biofilm Elimination Concentrations.

**Table 2:** Percent mortality of *Artemia salina* larvae in increasing concentrations of solutions with ORLE and AgNPs(ORLE).

Tested Concentration (μg/mL)	% Mortality	
	ORLE	AgNPs(ORLE)
150	4.0 ± 3.3	7.1 ± 3.4
200	33.9 ± 9.9	36.6 ± 11.6
250	34.5 ± 14.1	26.4 ± 11.4
300	78.4 ± 13.4	78.8 ± 23.1

## Supplementary Materials

The following are available online at https://www.mdpi.com/article/10.3390/ijms22073539/s1. Figure S1. Dry discs of hydrogels pHEMA@ORLE_2 (left) and pHEMA@AgNPs(ORLE)_2 (right). Figure S2. Bacterial growth inhibition zones developed in *P. aeruginosa* (A), *E. coli* (B), *S. epidermidis* (C), and *S. aureus* (D) by ORLE and AgNPs(ORLE) with doses of 1 or 2 mg/mL upon their incubation for 20 h. Figure S3. Bacterial growth inhibition zones developed in *P. aeruginosa* (A), *E. coli* (B), *S. epidermidis* (C), and *S. aureus* (D) by ORLE and pHEMA@ORLE with doses of 1 or 2 mg/mL upon their incubation for 20 h. Figure S4. Removal of preformed biofilm of *P. aeruginosa* (A) and *S. aureus* (B) caused pHEMA, pHEMA@ORLE_2, and pHEMA@AgNPs(ORLE)_2 discs. Figure S5. Minimum inhibitory concentration of ORLE (A) and AgNPs(ORLE) (B) against *P. aeruginosa*. Figure S6. minimum inhibitory concentration of ORLE (A) and AgNPs(ORLE) (B) against *E. coli*. Figure S7. Minimum inhibitory concentration of ORLE (A) and AgNPs(ORLE) (B) against *S. epidermidis*. Figure S8. Minimum inhibitory concentration of ORLE (A) and AgNPs(ORLE) (B) against *S. aureus.* Figure S9. Minimum bactericidal concentration of AgNPs(OLRE) against *P. aeruginosa* (A), *E. coli* (B), *S. epidermidis* (C), and *S. aureus* (D). Figure S10. Minimum bactericidal concentration of ORLE against *P. aeruginosa* (A), *E. coli* (B), *S. epidermidis* (C), and *S. aureus* (D). Figure S11. The biofilm growth of *P. aeruginosa* (A) and *S. aureus* (B), under increasing concentrations of AgNPs(ORLE) stained by crystal violet.

## Abbreviations

ATR–FT–IR	Attenuated Total Reflection mode
ΒΕC	Biofilm Elimination Concentration
DTG/DSC	Differential Scanning Calorimetry
*E. coli*	*Escherichia coli*
FWHM	Full Width at Half Maximum
HCEC	Normal Human Corneal Epithelial Cells
IZ	Inhibitory Zone
MBC	Minimum Bactericidal Concentration
MIC	Minimum Inhibitory Concentration
MK	Microbial Keratitis
*P. aeruginosa*	*Pseudomonas aeruginosa*
pHEMA	poly–2–hydroxyethylmethacrylate
*S. aureus*	*Staphylococcus aureus*
*S. epidermidis*	*Staphylococcus epidermidis*
TD–DTA	Thermogravimetric Differential Thermal Analysis
UV–Vis	Ultra violet
XRF	X-ray fluorescence spectroscopy
XRPD	X-ray powder diffraction analysis
