# Supplementary material for: Silver Nanoparticles from Oregano Leaves’ Extracts as Antimicrobial Components for Non-Infected Hydrogel Contact Lenses
Source: Int J Mol Sci. 2021 Mar 29;22(7):3539. doi: 10.3390/ijms22073539 (PMC8037402; doi:10.3390/ijms22073539)
Supplement: Supplementary file 1 [file ijms-22-03539-s001.pdf]

International Journal of *Molecular Sciences*

MDPI

Article

Supplementary Information

Silver Nanoparticles from Oregano Leaves' Extracts as Antimicrobial Components for Non-Infected Hydrogel Contact Lenses

A. Meretoudi [1], C.N. Banti [1,*], P.K. Raptis [1], C. Papachristodoulou [2], N. Kourkoumelis [3], A.A. Ikiades [2], P. Zoumpoulakis [4], T. Mavromoustakos [5] and S.K. Hadjikakou [1,6,*]

[1] Inorganic Chemistry laboratory, Department of Chemistry, University of Ioannina, 45110, Ioannina, Greece; ameretoudi1996@gmail.com, cbanti@uoi.gr, shadjika@uoi.gr
[2] Department of Physics, University of Ioannina, Greece; xpapaxri@uoi.gr; ikiadis@uoi.gr
[3] Medical Physics Laboratory, Medical School, University of Ioannina, Greece; nkourkou@uoi.gr
[4] Laboratory of Chemistry, Analysis & Design of Food Processes, Department of Food Science and Technology, University of West Attica, Greece; pzoump@eie.gr
[5] Organic Chemistry Laboratory, Department of Chemistry, University of Athens Greece; tmavrom@chem.uoa.gr
[6] University Research Center of Ioannina (URCI), Institute of Materials Science and Computing, Ioannina, Greece; shadjika@uoi.gr
* Correspondence: shadjika@uoi.gr (SKH); Tel.: +30-26510-08374 (SKH)

Citation: Meretoudi, A.; Banti, C.N.; Raptis, P.K.; Papachristodoulou, C.; Kourkoumelis, N.; Ikiades, A.A.; Zoumpoulakis, P.; Mavromoustakos, T.; Hadjikakou, S.K.; Silver Nanoparticles from Oregano Leaves Extracts as Antimi-crobial Components for Non-Infected Hydrogel Contact Lenses *Int. J. Mol. Sci.* **2021**, *22*, x. https://doi.org/10.3390/xxxxx

Academic Editor: Iolanda Francolini

Received: 12 March 2021
Accepted: 25 March 2021
Published: 29 March 2021

Publisher's Note: MDPI stays neutral with regard to jurisdictional claims in published maps and institutional affiliations.

Copyright: © 2021 by the authors. Submitted for possible open access publication under the terms and conditions of the Creative Commons Attribution (CC BY) license (http://creativecommons.org/licenses/by/4.0/).

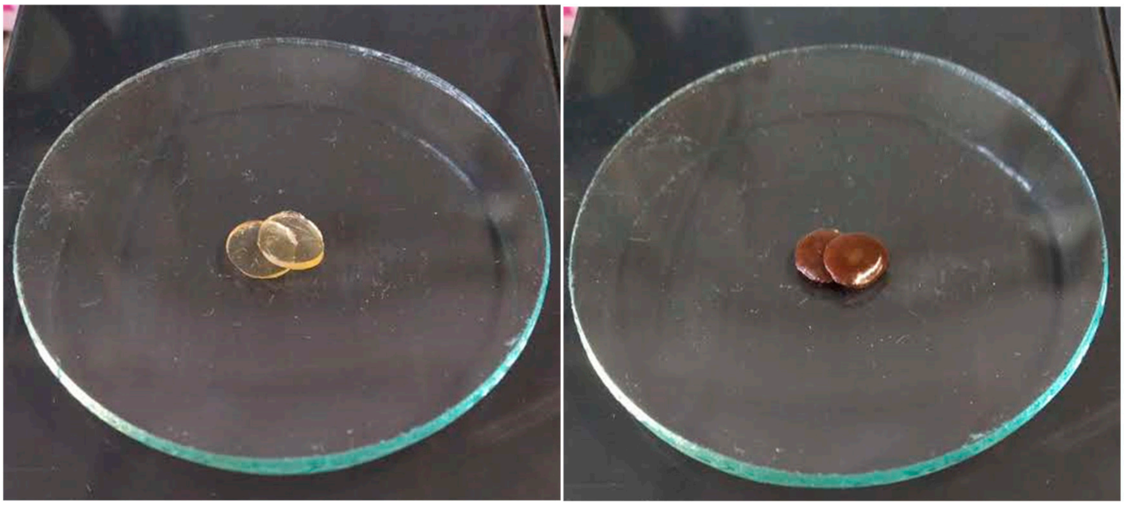

Figure S1. Dry discs of hydrogels pHEMA@ORLE_2 (**left**) and pHEMA@AgNPs(ORLE)_2 (**right**).

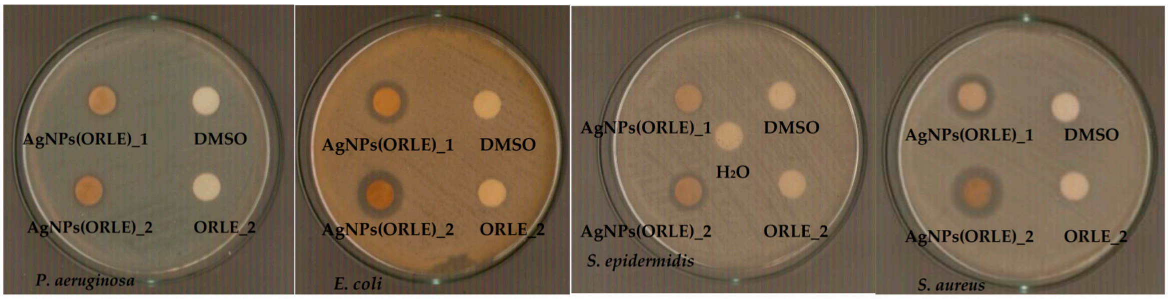

[A] [B] [C] [D]

Figure S2. Bacterial growth inhibition zones developed in *P. aeruginosa* (***A***), *E. coli* (***B***), *S. epidermidis* (***C***), and *S. aureus* (**D**) by ORLE and AgNPs(ORLE) with doses of 1 or 2 mg/mL upon their incubation for 20 h.

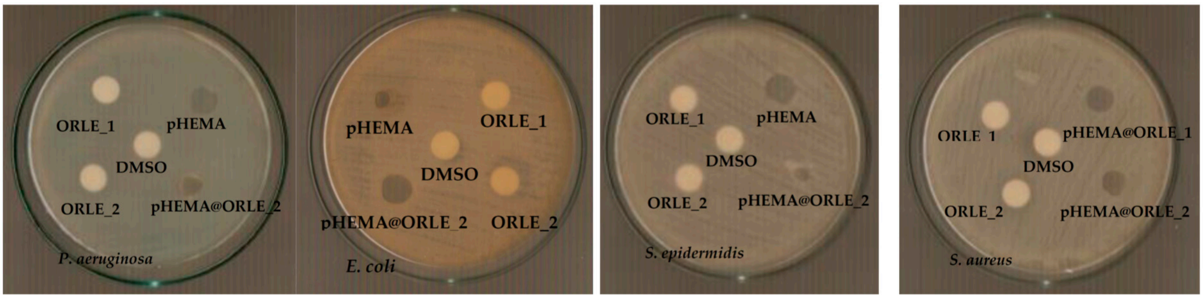

[A] [B] [C] [D]

Figure S3. Bacterial growth inhibition zones developed in *P. aeruginosa* (***A***), *E. coli* (***B***), *S. epidermidis* (***C***), and *S. aureus* (**D**) by ORLE and pHEMA@ORLE with doses of 1 or 2 mg/mL upon their incubation for 20 h.

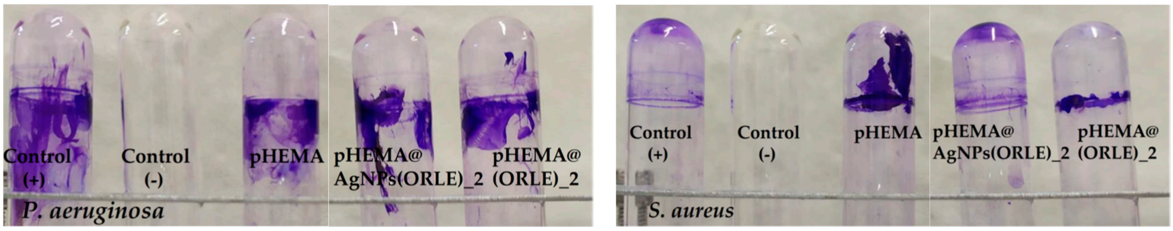

(A) (B)

Figure S4. Removal of preformed biofilm of *P. aeruginosa* (**A**) and *S. aureus* (**B**) caused pHEMA, pHEMA@ORLE_2, and pHEMA@AgNPs(ORLE)_2 discs.

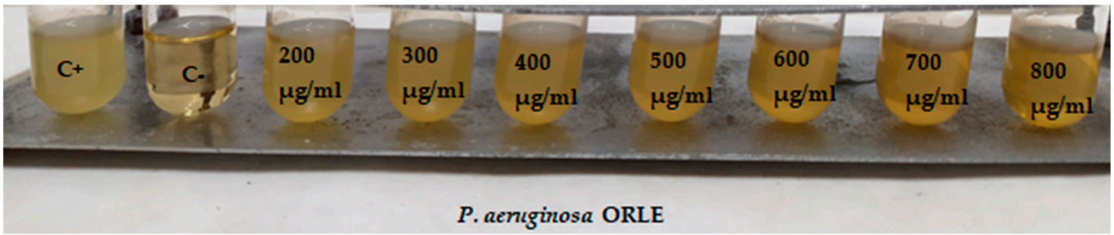

(A)

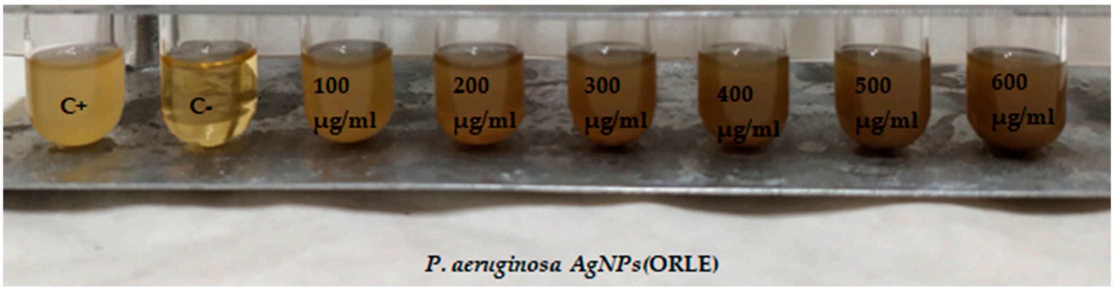

(B)

Figure S5. Minimum inhibitory concentration of ORLE **(A)** and AgNPs**(ORLE) (B)** against *P. aeruginosa*.

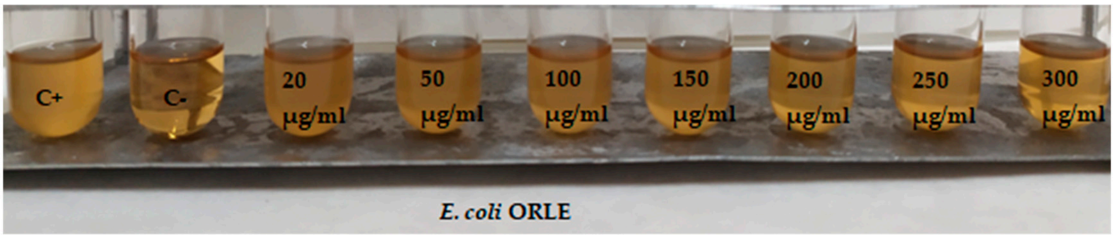

(A)

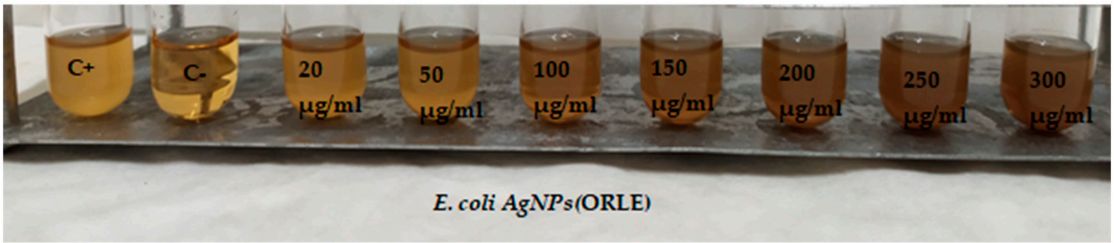

(B)

Figure S6. Minimum inhibitory concentration of ORLE **(A)** and AgNPs(ORLE) **(B)** against *E. coli.*

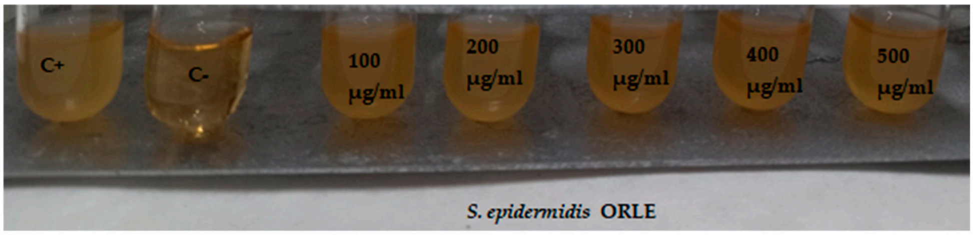

(A)

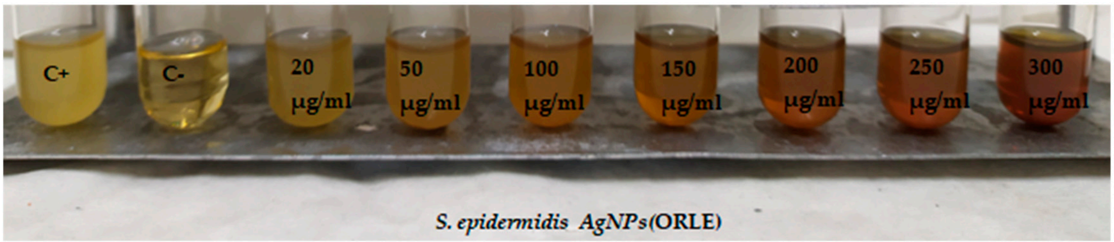

(B)

Figure S7. Minimum inhibitory concentration of ORLE **(A)** and AgNPs(ORLE) (**B**) against *S. epidermidis.*

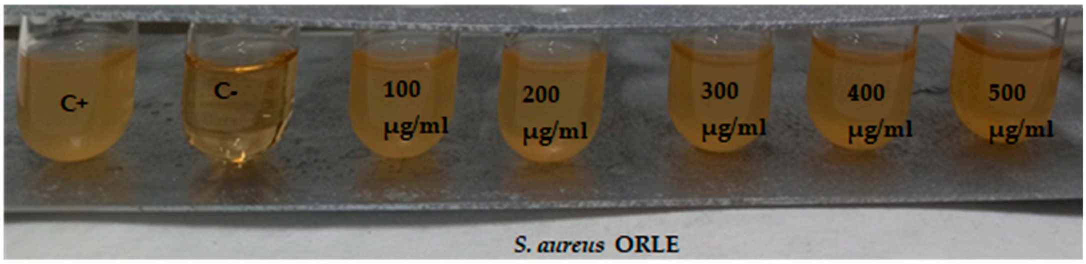

(A)

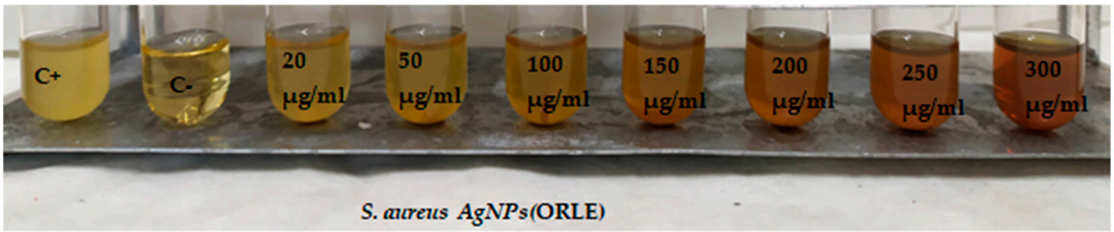

(B)

Figure S8. Minimum inhibitory concentration of ORLE **(A)** and AgNPs(ORLE) **(B)** against *S. aureus.*

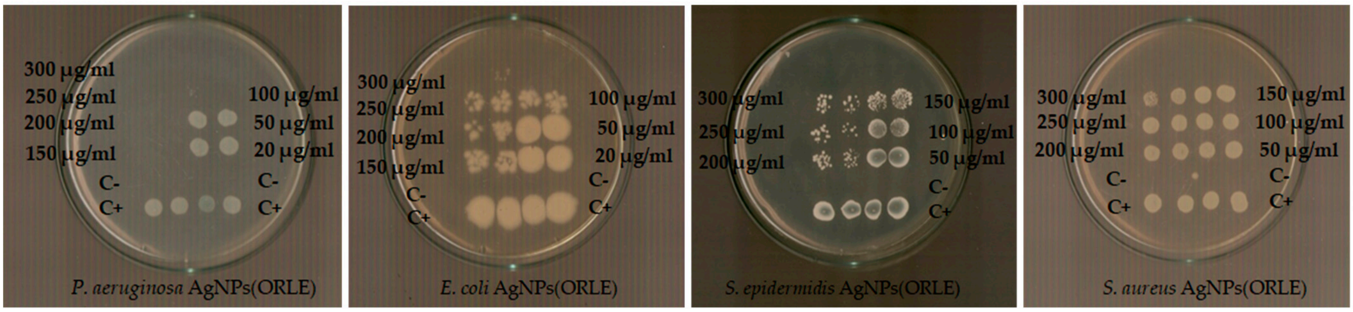

Figure S9. Minimum bactericidal concentration of AgNPs(OLRE) against *P. aeruginosa* (**A**), *E. coli* (**B**), *S. epidermidis* (**C**), and *S. aureus* (**D**).

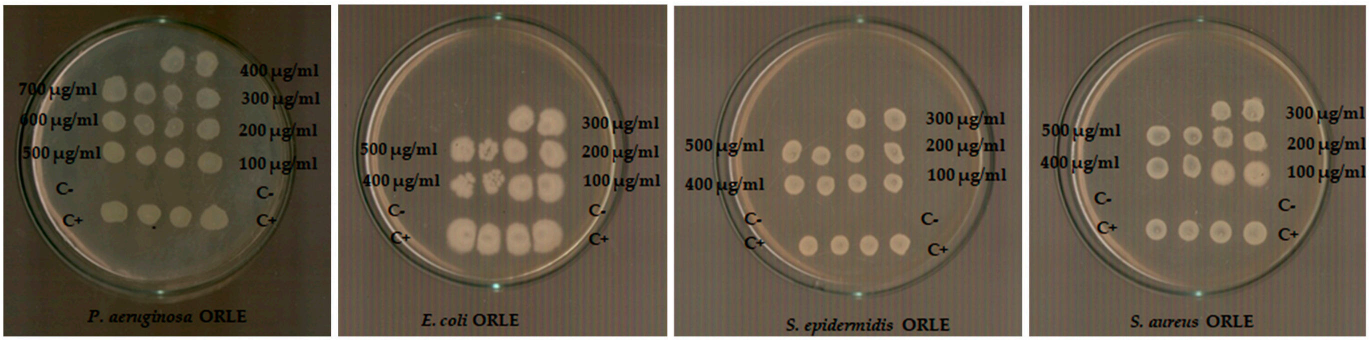

Figure S10. Minimum bactericidal concentration of OLRE against *P. aeruginosa* (A), *E. coli* (B), *S. epidermidis* (C), and *S. aureus* (D).

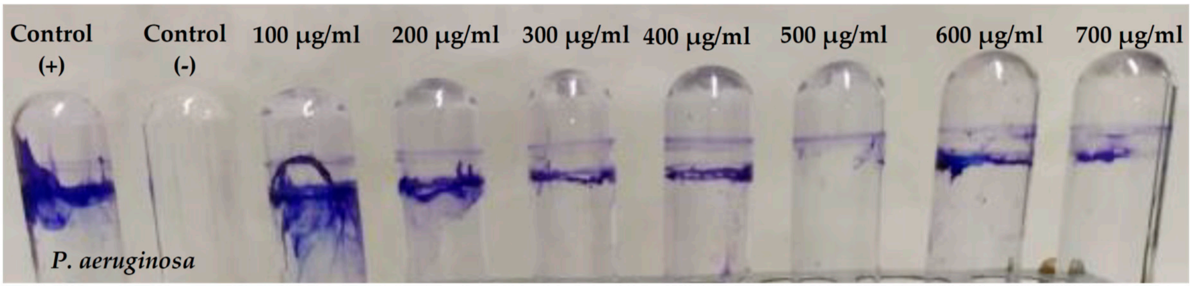

(A)

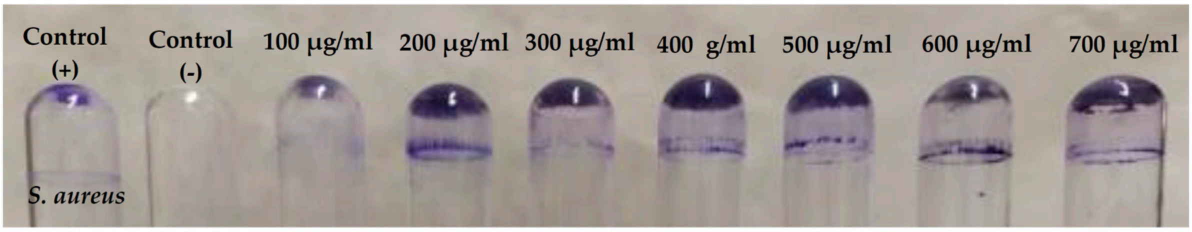

(B)

Figure S11. The biofilm growth of *P. aeruginosa* (A) and *S. aureus* (B), under increasing concentrations of AgNPs(ORLE) stained by crystal violet.
